# Managing Side Effects of Vemurafenib Therapy for Advanced Melanoma

**Published:** 2014-11-01

**Authors:** Brenda Hagen, Van Anh Trinh

**Affiliations:** University of Texas MD Anderson Cancer Center, Houston, Texas

## Abstract

Somatic point mutations in the *BRAF* gene have been found in approximately 50% of melanomas. *BRAF*^V600E^, the most common mutation, results in the constitutive activation of *BRAF*^V600E^ kinase, sustaining MAPK signaling and perpetuating cell growth. This groundbreaking discovery led to the clinical development of vemurafenib, a selective *BRAF* inhibitor. Vemurafenib has been approved for the treatment of patients with *BRAF*^V600E^-positive unresectable or metastatic melanoma based on survival benefit demonstrated in a randomized phase III study. The current approved dosing schedule of vemurafenib is 960 mg orally twice a day until disease progression or unacceptable toxicity. Vemurafenib is well tolerated, with the most common adverse effects including skin reactions, photosensitivity, headache, and arthralgia. Active research is ongoing to expand the utility of vemurafenib into the adjuvant setting and to circumvent rapid emergence of drug resistance.

The incidence of melanoma has been rising over the past 30 years. In 2014, it is estimated that 76,100 new cases will be diagnosed and 9,710 individuals will die of melanoma in the United States (Siegel et al., 2014). The prognosis for patients with advanced melanoma has been grim, with a 1-year survival rate of 25% and a median overall survival of 6.2 months (Korn et al., 2008). Until recently, therapeutic options for unresectable or metastatic melanoma have also been limited. For more than 2 decades, dacarbazine and interleukin-2 (IL-2) were the only agents approved by the US Food and Drug Administration (FDA) for the treatment of advanced or metastatic melanoma.

In 2011, the FDA approved iplimumab (Yervoy), a novel T-cell potentiator, for advanced melanoma. Ipilimumab blocks the inhibitory T-cell surface-molecule cytotoxic T-lymphocyte antigen-4, thus augmenting the T-cell response to melanoma antigens. Ipilimumab was the first agent shown to improve overall survival for patients with advanced melanoma in phase III studies. Also approved for the management of advanced melanoma in 2011 was vemurafenib (Zelboraf). Unlike ipilimumab, vemurafenib’s labeled indication is restricted to the subgroup of patients whose tumors tested positive for *BRAF*^V600E^ mutation. This review will focus on the pharmacology, pharmacokinetics, and side-effect management of vemurafenib in patients with advanced melanoma.

## MECHANISM OF ACTION

The mitogen-activated protein kinase (MAPK) pathway is an important signaling cascade regulating cell growth, differentiation, and survival (Figure Normally, MAPK pathway activation begins with the binding of extracellular growth factors, such as epidermal growth factor (EGF), to membrane-bound receptor tyrosine kinases (RTKs), such as epidermal growth factor receptor (EGFR; Chapman & Miner, 2011). Sequential phosphorylation of RAS, RAF, MEK, and ERK communicates the growth signal downstream to the nucleus, inducing gene expression to promote cell proliferation, differentiation, and survival.

**Figure 1:**
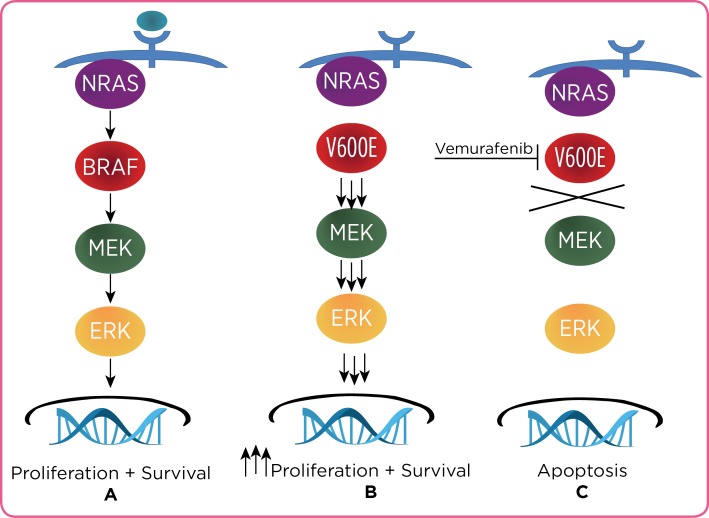
MAPK pathway and mechanism of vemurafenib. (A) When extracellular growth factor binds to membrane-bound receptor tyrosine kinase, MAPK signaling pathway is activated to promote cell proliferation and survival. (B) V600E kinase is constitutively active, sustaining MAPK signaling and perpetuating cell growth in the absence of growth factor. (C) Vemurafenib inhibits V600E kinase, inducing apoptosis.

A decade ago, melanoma tumors were found to harbor genetic mutations in various components of the MAPK signaling pathway. *BRAF* mutation is the most common event, occurring in about 50% of melanomas. The most frequent *BRAF* mutation is *V600E*, a point mutation resulting in the valine-to-glutamic acid substitution at amino acid 600. BRAF^V600E^ kinase is constitutively active, sustaining MAPK signaling and perpetuating cell growth (Davies et al., 2002). Phenotypically, BRAF^V600E^ confers aggressive behavior to melanoma cells (Klein & Alpin, 2009; Arozarena et al., 2011) and has been linked to an unfavorable survival outcome in patients with metastatic disease (Long et al., 2011). Additionally, there is evidence indicating a strong association between *BRAF* mutations and the frequency of central nervous system metastases at the time of stage IV diagnosis (Jakob et al., 2011).

Identification of activating *BRAF* mutations in melanoma tumors has fostered development of the selective small-molecule inhibitor of mutant BRAF kinase vemurafenib for the treatment of advanced melanoma. Blocking the mutation-driven constitutive MAPK pathway signaling, vemurafenib demonstrated marked antitumor activity in melanoma cell lines harboring the *BRAF*^V600E^ mutation. Recently, vemurafenib has been shown to improve overall survival for patients with *BRAF*^V600E^-positive advanced melanoma in a randomized phase III study that led to its FDA approval in 2011 (Chapman et al., 2011).

Besides mutant BRAF kinases, vemurafenib also inhibits wild-type (nonmutated) BRAF and CRAF (another RAF isoform) enzymes. Interestingly, vemurafenib can paradoxically activate the MAPK pathway via heterodimerization of *BRAF* and CRAF kinases to promote growth in tumors harboring wild-type *BRAF* or activating *RAS* mutations. Therefore, vemurafenib monotherapy should not be used in patients with advanced melanoma with wild-type *BRAF* or *RAS* mutation (Heidorn et al., 2010; Poulikakos, Zhang, Bollag, & Shokat, 2010; Hatzivassiliou et al., 2010).

## PHARMACOKINETICS

Current pharmacokinetic data are based on a pooled analysis of 458 patients with *BRAF* mutation–positive advanced melanoma following 15 days of vemurafenib at 960 mg twice daily. (Genentech, 2014). The bioavailability of vemurafenib has not been determined; however, its microprecipitated powder formulation has been deemed highly bioavailable (Flaherty et al., 2010). Based on experience in clinical trials, vemurafenib can be administered with or without food, although the effect of food on its absorption has not been studied. Time to maximum concentration is achieved about 3 hours postdose. Drug exposure increases proportionally with doses up to 960 mg twice daily. Steady state condition is reached between 15 and 22 days following treatment initiation, with a median elimination half-life of 57 hours, systemic clearance of 31 L/day, and volume of distribution of 106 L. Other variables such as age, gender, and body weight appeared to have no significant impact on vemurafenib clearance (Genentech, 2014).

Vemurafenib is primarily excreted via the feces. Dose adjustment is not needed for preexisting mild to moderate renal or hepatic dysfunction (creatinine clearance> 29 mL/min or total bilirubin 1 to 3 times the upper limit of normal, respectively), However, vemurafenib should be used with caution in the presence of severe liver or kidney impairment. In vitro studies with human hepatic microsomes demonstrated that vemurafenib is a substrate of cytochrome P450 (CYP) 3A4 and an inhibitor of several CYP enzyme systems, suggesting potential interactions with CYP3A4 inhibitors or inducers and other CYP substrates with narrow therapeutic index (Genentech, 2014).

## CLINICAL TRIAL RESULTS

**BRIM 1**

The BRAF Inhibitor in Melanoma 1 (BRIM 1) was a two-stage phase I dose-finding study of PLX4032, later known as vemurafenib. The trial began with a dose-escalation phase in 55 patients with metastatic cancer of various tumor types to define the safety profile and to establish the maximum tolerated dose (MTD) of vemurafenib. *BRAF* mutational status was not required for enrollment into this portion of the trial. Midway through the dose-escalation phase, the formulation of vemurafenib was switched from the initial poorly bioavailable crystalline preparation to the highly bioavailable microprecipitated bulk powder. With the improved formulation, dose-limiting toxicities, manifesting as grade 3 fatigue, rash, and arthralgia, occurred at the vemurafenib dose of 1,120 mg twice daily. The MTD or the recommended phase II dose (RP2D) was therefore set at 960 mg twice daily.

Upon determination of the RP2D, the extension phase followed to evaluate the response rate of vemurafenib in patients with metastatic melanoma whose tumors tested positive for *BRAF^V600E^* mutation. Of 32 patients, 26 (81%) achieved an objective response, most of them partial. Tumor regressions were observed across the patient population, even in those with poor-risk features, such as visceral organ involvement or an elevated level of lactate dehydrogenase. The onset of response seemed to be early, with symptomatic improvement noted within 1 or 2 weeks after treatment initiation. The duration of response ranged from 2 to 18 months, with a median progression-free survival of 7 months or more (Flaherty et al., 2010).

**BRIM 2**

The BRIM 2 trial was a phase II study conducted to verify the response rate to vemurafenib in previously treated patients with *BRAF^V600E^*–positive stage IV melanoma. A total of 132 patients were enrolled and treated with vemurafenib at 960 mg twice daily. The overall response rate was 53%, comprising 6% complete responses and 47% partial responses. At a median follow-up of 12.9 months, the median progression-free survival and overall survival were 6.7 months and 15.9 months, respectively. The toxicity profile of vemurafenib was consistent with previous experience from the BRIM 1 study, with the commonly reported adverse events consisting of skin rash, photosensitivity reaction, arthralgia, and fatigue (Sosman et al., 2012).

**BRIM 3**

The BRIM 3 study is the pivotal randomized phase III trial conducted to confirm the clinical benefit of vemurafenib. In this trial, 675 previously untreated patients with *BRAF*^V600E^-positive advanced melanoma were randomly assigned to receive vemurafenib 960 mg orally twice daily or dacarbazine 1 g/m² intravenously every 3 weeks. The rates of progression-free and overall survival were the primary endpoints of this study.

At the interim analysis at a median follow-up of 3.8 months for the vemurafenib-treated patients and 2.3 months for those given dacarbazine, vemurafenib was associated with a 63% relative reduction in the risk of death and a 74% relative reduction in the risk of disease progression compared with dacarbazine (*p* < .001). Other clinical benefits of vemurafenib included more rapid disease control and higher response rate (Chapman et al., 2011). Considering the impressive clinical benefit associated with vemurafenib, the independent data and monitoring board recommended allowing patients to cross over from the dacarbazine group to receive vemurafenib at disease progression. Median overall survival was not available at the interim analysis due to the short follow-up.

Survival results have recently been updated at a median follow-up of 12.5 and 9.5 months for the vemurafenib and dacarbazine arms, respectively. The median overall survival was 13.6 months in the vemurafenib group vs. 9.7 months in the dacarbazine group, with a hazard ratio for death of 0.70 (*p* = .0008) favoring vemurafenib. Median progression-free survival was significantly longer in the vemurafenib group than in the dacarbazine group (6.9 vs. 1.6 months; *p* < .0001; McArthur et al., 2014).

## PATIENT SELECTION, DOSING, AND ADMINISTRATION

Since vemurafenib is indicated for the treatment of patients with *BRAF^V600E^*-positive unresectable or metastatic melanoma, the patient selection process should begin with *BRAF* mutational analysis of patients’ tumor tissues. The cobas 4800 BRAF V600 Mutation Test is the FDA-approved companion test for the qualitative detection of *BRAF*^V600E^ mutation in DNA extracted from formalin-fixed, paraffin-embedded human melanoma tissue. Once *BRAF*^V600E^ positivity is confirmed, the standard screening procedure includes a thorough dermatology exam, 12-lead electrocardiography, and liver function test. A complete review of concurrent medications to identify potential drug interactions with vemurafenib should also be conducted (Genentech, 2014).

Vemurafenib is available only through select specialty pharmacies. The time from prescription issuance to drug attainment can vary significantly depending on third-party payers’ procedures for authorization. However, enrolling patients to the Zelboraf Access Solutions program (www.genentech-access.com/zelboraf/patients) may help bridge the coverage delay, expedite drug procurement, and provide financial assistance.

Vemurafenib, available as a 240-mg tablet, is dosed at 960 mg orally twice daily with or without a meal. Patients should be instructed to swallow the tablets whole with a glass of water. If a dose is missed, it can be made up to maintain the twice-daily regimen as long as there is a minimum of 4 hours from the next scheduled dose.

Currently, vemurafenib therapy is continued without a break until disease progression or unacceptable toxicity. However, an intermittent dosing schedule is being considered to deter the emergence of drug resistance (Das Thakur et al., 2013). Indeed, tumor sensitivity to selective BRAF inhibitors such as vemurafenib was restored after a drug-free period in two patients with *BRAF*^V600E^-mutant advanced melanoma who had previously developed disease progression during therapy with selective BRAF inhibition (Seghers, Wilgenhof, Lebbe, & Neyns, 2012).

## DOSE-MODIFICATION RECOMMENDATIONS

Vemurafenib is generally well tolerated. In the BRIM 3 study, 38% of patients required dose modification or interruption due to adverse events; however, permanent treatment discontinuation occurred in only 7% of the study population (Chapman et al., 2011).

Vemurafenib dose-modification guidelines are shown in Table 1. It is recommended that vemurafenib be interrupted for intolerable grade 2 or 3 toxicities. Once adverse events have subsided to grade 0 or 1, vemurafenib can be resumed at 720 mg twice daily. For the second appearance of intolerable grade 2 or 3 side effects, the dose of vemurafenib should be further reduced to 480 mg orally twice daily. If more than two dose reductions are required, vemurafenib should be permanently discontinued.

**Table 1:**
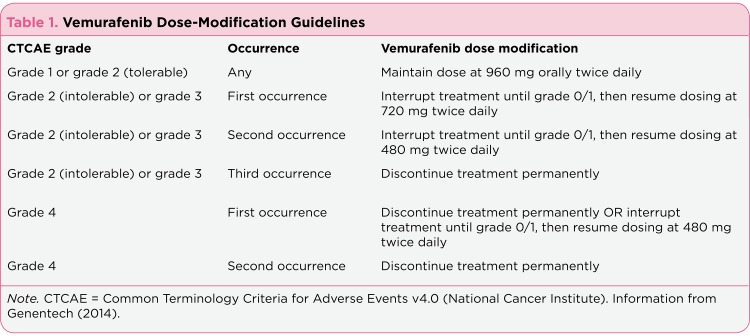
Vemurafenib Dose-Modification Guidelines

The occurrence of grade 4 toxicities necessitates more drastic dose modification. Upon resolution of the first appearance of grade 4 adverse events, vemurafenib should be restarted at 480 mg twice daily. For the second appearance of a grade 4 adverse event, vemurafenib should be permanently discontinued (Genentech, 2014).

## MANAGEMENT OF SPECIFIC TOXICITIES

Table 2 lists management options for specific toxicities associated with the use of vemurafenib.

**Table 2:**
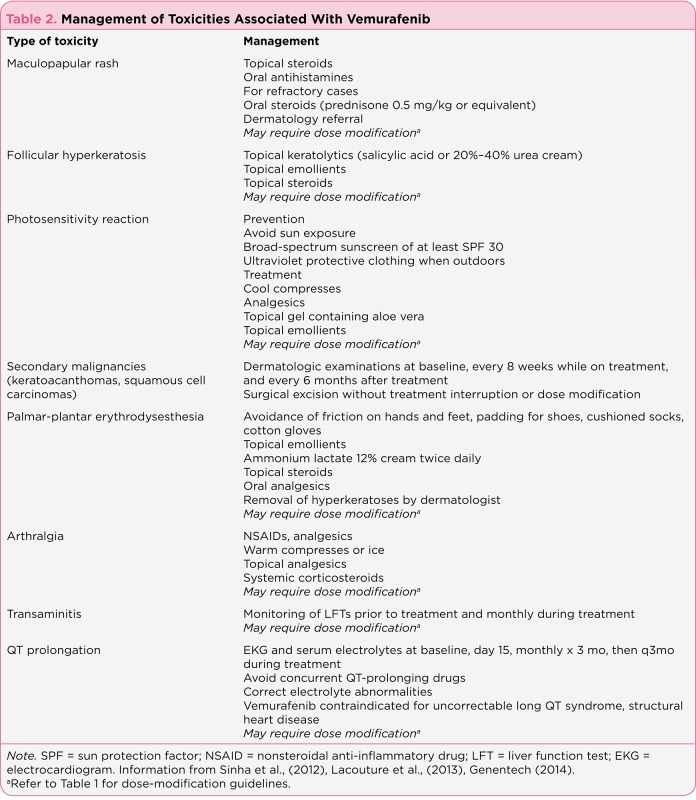
Management of Toxicities Associated With Vemurafenib

**Dermatologic Reactions**

Dermatologic manifestations made up a major part of vemurafenib’s toxicity profile (Flaherty et al., 2010; Sosman et al., 2011; Chapman et al., 2011). Thus, the Vemurafenib Dermatology Working Group (DWG), an expert panel including dermatologists, oncologists, and dermatopathologists, was asked to evaluate the composite cutaneous adverse event data from the BRIM 1, 2, and 3 studies to provide management guidelines for these toxicities (see Table 2; Lacouture et al., 2013). According to the DWG report, dermatologic reactions affected 92% to 95% of patients, with skin rash, photosensitivity, and cutaneous squamous cell carcinoma (cSCC) among the most frequently encountered.

*Skin Rash and Pruritus*: The overall incidence of skin rash, mostly of grade 1 or 2, was 64% to 75% (Lacouture, 2013). Grade 3 rash was uncommon, affecting 8% of patients. The spectrum of rash encompassed many subtypes, such as maculopapular, follicular, erythema, keratosis-pillaris–like, or not otherwise specified eruptions (Lacouture et al., 2013). Skin rash typically occurred early, with median time to onset of 1.6 weeks from vemurafenib initiation (Lacouture et al., 2013). The rash affected the face, neck, trunk and extremities (Flaherty, 2010), and was variably associated with itching or soreness (Huang, Hepper, Anadkat, & Cornelius, 2012; Rinderknecht et al., 2013). The development or severity of rash did not seem to correlate with tumor response (Lacouture et al., 2013).

Examination of skin rash biopsies did not reveal a consistent histopathologic pattern to pinpoint a specific mechanism. In those specimens, the presence of perifollicular or perivascular inflammatory infiltrates comprising lymphocytes and eosinophils resembled drug-induced eruptions. Nevertheless, the fact that most patients with skin rash were able to continue vemurafenib with or without dose reduction argued against a true hypersensitivity reaction (Lacouture et al., 2013; Sinha et al., 2012).

Patients experiencing a rash should have a physical examination for accurate assessment. The main management approach for maculopapular rash begins with topical steroids and oral antihistamines. Refractory cases may require systemic steroids, vemurafenib interruption and referral to dermatologists. For follicular hyperkeratotic eruptions, keratolytic agents such as salicylic acid or urea cream should accompany topical steroids and oral antihistamines. Fortunately, most skin rash improved with supportive care and did not require vemurafenib dose modification or interruption (Lacouture et al., 2013).

*Photosensitivity*: Photosensitivity was experienced in 35% to 63% of patients receiving vemurafenib, with 3% of them grade 3 or higher (Lacouture et al., 2013). Photosensitivity reactions developed early, with a median time to appearance of 1.7 weeks from therapy initiation, and occurred within 24 hours from sun exposure, even when solar intensity appeared minimal. Vemurafenib-related phototoxicity is ultraviolet A (UVA)-dependent and can be prevented with a broad-spectrum sunscreen (Dummer et al., 2012). Thus, prior to the commencement of vemurafenib, patients should be advised to avoid sun exposure and to use a broad-spectrum sunscreen and ultraviolet-protective clothing when outdoors.

Patients should be counseled regarding the proper selection of sunscreen products. Appropriate sunscreen products should have a sun protection factor (SPF) of at least 30 and should cover the UVA wavelength (Dummer et al., 2012; Sinha et al., 2012). Active ingredients conferring UVA protection are avobenzone, ecamsule, zinc oxide and titanium dioxide (Sinha et al., 2012).

The proper usage of sunscreen should also be demonstrated and emphasized. A liberal amount (at least 1 ounce) of sunscreen should be applied to sun-exposed areas at least 30 minutes before going outside. Reapplication of sunscreen should be repeated every 2 hours and immediately after swimming or sweating profusely. In addition, patients should be advised about the distinct characteristics of UVA rays, such as their constant intensity despite the season and daytime and their ability to penetrate glass (Dummer et al., 2012).

*Keratoacanthoma and Squamous Cell Carcinoma of the Skin*: A distinct feature of the *BRAF* inhibitors is their ability to induce secondary cutaneous malignancies, such as keratoacanthomas (KAs) and cSCCs. Keratoacanthomas are cutaneous lesions typically seen on sun-exposed areas of the body. Most KAs present as solitary, dome-shaped nodules with central keratin-filled crateriform ulceration (Mandrell & Santa Cruz, 2009). Their clinical course is characterized by a rapid growth phase followed by a gradual period of regression, which leads to their spontaneous resolution within a few months. These KAs are considered to be benign squamous cell proliferations, although that remains debatable, as rare cases of invasive or metastasizing KAs have been reported. Due to this uncertainty, the standard management of KAs is surgical resection (Mandrell & Santa Cruz, 2009).

Keratoacanthomas and cSCCs developed on sun-exposed skin in 19% to 26% of patients treated with vemurafenib in the BRIM studies, with a median time to first occurrence of 7.1 weeks after vemurafenib initiation (Lacouture et al., 2013). Patients may have multiple cSCC lesions, and the median time between the first and the second one was approximately 6 weeks (Sinha et al., 2012).

Vemurafenib-induced squamoproliferative skin lesions shared the same risk factors as sporadic cSCCs: age 65 or older and chronic sun exposure. The proposed mechanism underlying the development of cSCCs is that *BRAF* inhibitors paradoxically activate the MAPK pathway in cutaneous squamous cells harboring *HRAS* mutations, which are present in 20% of squamous skin tissues (Oberholzer et al., 2012; Su et al., 2012). If detected, these skin cancers should be completely excised without treatment modification or interruption.

Thorough dermatologic examination is recommended at baseline, every 8 weeks during vemurafenib therapy, and for 6 months following drug discontinuation (Genentech, 2014). Patients should be instructed to perform regular skin self-examination and to report any nonhealing lesions, changes to existing moles, or new skin eruptions.

*Palmar-Plantar Erythrodysesthesia*: Palmar-plantar erythrodysesthesia (PPE) was reported in 8% to 10% of patients on vemurafenib (Lacouture, 2013) and usually presented as painful, yellow, hyperkeratotic calluses at points of friction or pressure surrounded by erythema on the palms and soles, with the soles more commonly affected (Boussemart et al., 2013). Patients should be instructed to practice preventative measures, including heavy use of topical emollients and avoidance of friction on hands and feet.

Once developed, PPE should be managed with topical keratolytic preparations such as urea 20% to 40% cream and symptom support with rest, cool compresses, topical emollients, and topical steroids. Oral analgesics can be added for pain and discomfort (Lacouture et al., 2013). If the condition does not improve, vemurafenib suspension is warranted.

*Other Skin Manifestations*: Other common dermatologic side effects include alopecia, dry skin, and skin papilloma. Vasculitis, erythema nodosum, and panniculitis were infrequently observed with the use of vemurafenib and typically managed with nonsteroidal anti-inflammatory drugs (NSAIDs) or a short course of oral corticosteroids for symptom relief (Lacouture et al., 2013). Serious and potential life-threatening conditions such as Stevens-Johnson syndrome or toxic epidermal necrolysis are rare but have been documented (Lacouture et al., 2013).

In addition to squamoproliferative lesions, new dysplastic nevi and primary melanomas have been reported in patients on vemurafenib (Dalle, Poulalhon, & Thomas, 2011; Zimmer et al., 2012), emphasizing the need for routine skin exams and close collaboration with dermatologists when monitoring vemurafenib therapy. Exaggerated radiodermatitis has recently been reported in patients receiving radiotherapy and vemurafenib (Satzger et al., 2013; Anker et al., 2013). It is recommended that vemurafenib be withheld for 7 days pre- and postradiotherapy (Anker et al., 2013). Patients should also be monitored closely for radiation recall when vemurafenib is resumed after radiation (Anker et al., 2013).

**Arthralgia**

The incidence of arthralgia, characterized by marked discomfort in one or more joints, was seen in 59% and 49% of patients in the BRIM 2 and BRIM 3 studies, respectively. Grade 3 events were seen in 6% of patients in the BRIM 2 trial and 3% in the BRIM 3 trial (Sosman et al., 2012; Chapman et al., 2011). The severity of joint pain can range from mild to debilitating, and the duration of symptoms can vary widely. The goal of treatment is symptom management, which relies primarily on the use of NSAIDs. Application of warm compresses or ice to affected joints, warm baths and topical analgesics may be beneficial. Arthralgia that is severe and debilitating may require a short course of systemic corticosteroids and vemurafenib interruption.

**Hepatotoxicity**

Transaminitis and hepatotoxicity have been reported with patients receiving vemurafenib. Elevated liver enzymes were reported in 17% of the study population in the BRIM 2 trial, with grade 3 abnormalities seen in 6% of patients (Sosman et al., 2012). Therefore, liver function tests (LFTs) should be monitored before treatment initiation and monthly during vemurafenib therapy. Elevated LFTs of grade 3 or higher should be managed with vemurafenib suspension followed by dose reduction. Recurrent hepatotoxicity despite dose reduction warrants vemurafenib discontinuation. In fact, the additive hepatotoxicity seen with concurrent administration of vemurafenib and ipilimumab in a phase I study led to termination of the trial (Ribas, 2013).

**QT Prolongation**

A potentially serious side effect of vemurafenib is QT prolongation, which predisposes patients to torsade de pointes, syncope, seizures, and sudden cardiac death. Electrocardiography and serum electrolytes should be evaluated prior to vemurafenib initiation, on day 15, monthly for 3 months, and every 3 months thereafter for the treatment duration. Vemurafenib should be avoided in patients with uncorrectable long QT syndrome (e.g., a congenital disorder or structural heart disease). In the presence of acquired QT prolongation secondary to electrolyte abnormalities or concomitant administration of QT-prolonging drugs, vemurafenib should be withheld until underlying causes are corrected. During therapy, vemurafenib should be interrupted if the QTc exceeds 500 msec and should be reinitiated at a reduced dose once the QTc is below 500 msec. After other risk factors for QT prolongation are eliminated, if the QTc remains longer than 500 msec with an absolute change from pretreatment value of more than 60 msec, vemurafenib should be discontinued permanently (Genentech, 2014).

**Secondary Malignancies**

Because vemurafenib paradoxically activates the MAPK pathway in select tissues, concern about secondary malignancies with this agent extends past cutaneous neoplasms. In fact, a case of vemurafenib-induced accelerated expansion of preexisting NRAS-mutant leukemic clones has been reported (Callahan et al., 2012). Occurrence of invasive SCC of the vulva and development of multiple premalignant colonic and gastric adenomas have also been described in patients with *BRAF*-mutant melanoma treated with vemurafenib (Boussemart et al., 2013; Gibney et al., 2013). Altogether, these observations emphasize the need for careful monitoring and heightened suspicion for secondary malignancies during vemurafenib therapy.

## DRUG RESISTANCE AND FUTURE DIRECTIONS

Despite the impressive tumor response and survival impact associated with vemurafenib in patients with advanced melanoma, its duration of response is short, implicating rapid emergence of drug resistance. Intriguingly, unlike other small-molecule kinase inhibitors, secondary mutations to the drug-binding domain of BRAF^V600E^ kinase have not yet been identified. The mechanisms of tumor resistance to selective BRAF inhibitors are diverse and complex; however, they frequently share a common endpoint—MAPK pathway reactivation (Sullivan, 2013). Measures to circumvent drug resistance are being identified and evaluated in animal models and ongoing clinical trials.

One approach to delay the development of drug resistance and enhance tumor response is to combine a selective BRAF inhibitor with other targeted agents. Since MAPK pathway reactivation is a common theme of tumor-escape mechanisms with BRAF inhibitors, the combination of a BRAF inhibitor and a MEK inhibitor has been evaluated and shown to generate superior benefit in prolonging progression-free survival and reducing cutaneous adverse events in BRAF inhibitor-naive patients (Long et al., 2014). In fact, in the coBRIM trial, a randomized, double-blind, placebo-controlled phase III study, the combination of vemurafenib and cobimetinib, a MEK inhibitor, was associated with a significant improvement in progression-free survival in previously untreated patients with *BRAF*^V600E^-mutated advanced melanoma when compared with vemurafenib monotherapy (Larkin et al., 2014).

Another strategy to improve efficacy and overcome drug resistance is to combine *BRAF* inhibitors with immunotherapy such as ipilimumab. Unfortunately, the phase I study examining the combination of ipilimumab and vemurafenib was immaturely terminated due to a high rate of hepatotoxicity (Ribas, 2013). Investigators are now assessing the toxicity profile of sequencing vemurafenib and ipilimumab in a phase II study (NCT01673854).

Expansion of the use of vemurafenib into the adjuvant setting is ongoing. A large phase III trial, BRIM 8, is being conducted to explore the safety and efficacy of single-agent vemurafenib as adjuvant therapy in patients with *BRAF*-mutant resected high-risk melanoma (NCT01667419).

## ROLE IN THERAPY FOR ADVANCED MELANOMA

The past 3 years have witnessed the addition of four new agents—ipilimumab, vemurafenib, dabrafenib (Tafinlar), and trametinib (Mekinist)—to the therapeutic repertoire for advanced melanoma, two of which are selective BRAF kinase inhibitors. In the absence of a direct comparison among these agents, it is difficult to determine which agent should be used first. The current consensus suggests that a selective BRAF inhibitor be used upfront in patients with mutant BRAF and rapidly progressive disease or in those requiring immediate relief of symptoms, as BRAF inhibitors typically induce a response more quickly than ipilimumab. At the present, there is no concrete evidence to suggest vemurafenib is superior to dabrafenib. Recognition of subtle differences between the BRAF inhibitors may be helpful in guiding therapy selection. Thus for now, therapy should be individualized based on the tumor’s *BRAF* mutational status, disease burden, performance status, and comorbidities (National Comprehensive Cancer Network, 2013).

Vemurafenib may expand the treatment option for patients with advanced melanoma involving the central nervous system. Despite confirmed clinical activity of vemurafenib against extracranial melanoma, data regarding the intracranial activity of vemurafenib are currently limited to a pilot study conducted by Dummer and colleagues (2014). In this study, 24 patients with *BRAF*^V600^ mutation–positive advanced melanoma and active brain metastases were treated with vemurafenib 960 mg orally twice daily. At enrollment, all patients required corticosteroid support and had failed to respond to at least one prior therapeutic modality for brain metastases. Half of the patients had four or more brain lesions, and 63% of them exhibited central nervous system–related symptoms at baseline.

The primary endpoint of this study was to assess the safety of vemurafenib in patients with active brain metastases. Secondary efficacy endpoints included best overall response rate, duration of response, progression-free survival, and overall survival, with best overall response rate calculated separately for intracranial, extracranial, and whole-body disease using RECIST (Response Evaluation Criteria in Solid Tumors) 1.1 criteria.

Confirmed intracranial partial response to vemurafenib was 16% (95% confidence interval [CI] = 3.4%–39.6%), with intracranial disease stabilization observed in 68% of patients (95% CI = 43.4%–87.4%). The median duration of tumor regression in the brain lasted 4.4 months (95% CI = 2.1–4.6 months). The overall response rate, based on tumor response with both intra- and extracranial disease, was 42% (95% CI = 22.1%–64.3%). The median progression-free survival and overall survival were 3.9 months (95% CI = 3.0–5.5 months) and 5.3 months (95% CI = 3.9–6.6 months), respectively.

Corresponding to the objective responses, patients’ symptomatology also improved, as evident by reduction in corticosteroid requirements, decrease in pain score, and improvement in performance status over baseline assessment. The safety profile of vemurafenib in this study was similar to previous experience from the other BRIM trials. One patient died of ileus occlusion; however, it was not thought to be related to treatment.

## CONCLUSION

Vemurafenib is the first FDA-approved BRAF inhibitor for the treatment of *BRAF*^V600E^ mutation–positive advanced melanoma. The adverse event profile associated with vemurafenib therapy can present clinical challenges; however, the majority of side effects are manageable with supportive intervention, dose modification, or treatment interruption. The mechanisms of tumor resistance to vemurafenib are being investigated and most likely relate to MAPK signaling reactivation. Clinical trials to expand the utility of vemurafenib into the adjuvant setting and to circumvent tumor resistance are currently in progress.

## References

[1] Anker Christopher J, Ribas Antoni, Grossmann Allie H, Chen Xinjian, Narra Krishna K, Akerley Wallace, Andtbacka Robert H I, Noyes Robert Dirk, Shrieve Dennis C, Grossmann Kenneth F (2013). Severe liver and skin toxicity after radiation and vemurafenib in metastatic melanoma.. *Journal of clinical oncology : official journal of the American Society of Clinical Oncology*.

[2] Arozarena Imanol, Sanchez-Laorden Berta, Packer Leisl, Hidalgo-Carcedo Cristina, Hayward Robert, Viros Amaya, Sahai Erik, Marais Richard (2011). Oncogenic BRAF induces melanoma cell invasion by downregulating the cGMP-specific phosphodiesterase PDE5A.. *Cancer cell*.

[3] Boussemart L, Routier E, Mateus C, Opletalova K, Sebille G, Kamsu-Kom N, Thomas M, Vagner S, Favre M, Tomasic G, Wechsler J, Lacroix L, Robert C (2013). Prospective study of cutaneous side-effects associated with the BRAF inhibitor vemurafenib: a study of 42 patients.. *Annals of oncology : official journal of the European Society for Medical Oncology / ESMO*.

[4] Callahan Margaret K, Rampal Raajit, Harding James J, Klimek Virginia M, Chung Young Rock, Merghoub Taha, Wolchok Jedd D, Solit David B, Rosen Neal, Abdel-Wahab Omar, Levine Ross L, Chapman Paul B (2012). Progression of RAS-mutant leukemia during RAF inhibitor treatment.. *The New England journal of medicine*.

[5] Chapman Mark S, Miner Jeffrey N (2011). Novel mitogen-activated protein kinase kinase inhibitors.. *Expert opinion on investigational drugs*.

[6] Chapman Paul B, Hauschild Axel, Robert Caroline, Haanen John B, Ascierto Paolo, Larkin James, Dummer Reinhard, Garbe Claus, Testori Alessandro, Maio Michele, Hogg David, Lorigan Paul, Lebbe Celeste, Jouary Thomas, Schadendorf Dirk, Ribas Antoni, O'Day Steven J, Sosman Jeffrey A, Kirkwood John M, Eggermont Alexander M M, Dreno Brigitte, Nolop Keith, Li Jiang, Nelson Betty, Hou Jeannie, Lee Richard J, Flaherty Keith T, McArthur Grant A (2011). Improved survival with vemurafenib in melanoma with BRAF V600E mutation.. *The New England journal of medicine*.

[7] Dalle S., Poulalhon N., Thomas L. (2011). Vemurafenib in melanoma with BRAF V600E mutation.. *New England Journal of Medicine*.

[8] Das Thakur Meghna, Salangsang Fernando, Landman Allison S, Sellers William R, Pryer Nancy K, Levesque Mitchell P, Dummer Reinhard, McMahon Martin, Stuart Darrin D (2013). Modelling vemurafenib resistance in melanoma reveals a strategy to forestall drug resistance.. *Nature*.

[9] Davies Helen, Bignell Graham R, Cox Charles, Stephens Philip, Edkins Sarah, Clegg Sheila, Teague Jon, Woffendin Hayley, Garnett Mathew J, Bottomley William, Davis Neil, Dicks Ed, Ewing Rebecca, Floyd Yvonne, Gray Kristian, Hall Sarah, Hawes Rachel, Hughes Jaime, Kosmidou Vivian, Menzies Andrew, Mould Catherine, Parker Adrian, Stevens Claire, Watt Stephen, Hooper Steven, Wilson Rebecca, Jayatilake Hiran, Gusterson Barry A, Cooper Colin, Shipley Janet, Hargrave Darren, Pritchard-Jones Katherine, Maitland Norman, Chenevix-Trench Georgia, Riggins Gregory J, Bigner Darell D, Palmieri Giuseppe, Cossu Antonio, Flanagan Adrienne, Nicholson Andrew, Ho Judy W C, Leung Suet Y, Yuen Siu T, Weber Barbara L, Seigler Hilliard F, Darrow Timothy L, Paterson Hugh, Marais Richard, Marshall Christopher J, Wooster Richard, Stratton Michael R, Futreal P Andrew (2002). Mutations of the BRAF gene in human cancer.. *Nature*.

[10] Dummer Reinhard, Goldinger Simone M, Turtschi Christian P, Eggmann Nina B, Michielin Olivier, Mitchell Lada, Veronese Luisa, Hilfiker Paul René, Felderer Lea, Rinderknecht Jeannine D (2014). Vemurafenib in patients with BRAF(V600) mutation-positive melanoma with symptomatic brain metastases: final results of an open-label pilot study.. *European journal of cancer (Oxford, England : 1990)*.

[11] Dummer Reinhard, Rinderknecht Jeannine, Goldinger Simone M (2012). Ultraviolet A and photosensitivity during vemurafenib therapy.. *The New England journal of medicine*.

[12] Flaherty Keith T, Puzanov Igor, Kim Kevin B, Ribas Antoni, McArthur Grant A, Sosman Jeffrey A, O'Dwyer Peter J, Lee Richard J, Grippo Joseph F, Nolop Keith, Chapman Paul B (2010). Inhibition of mutated, activated BRAF in metastatic melanoma.. *The New England journal of medicine*.

[13] (2014). Zelboraf (vemurafenib) package insert. *Genentech*.

[14] Gibney Geoffrey T, Messina Jane L, Fedorenko Inna V, Sondak Vernon K, Smalley Keiran S M (2013). Paradoxical oncogenesis--the long-term effects of BRAF inhibition in melanoma.. *Nature reviews. Clinical oncology*.

[15] Hatzivassiliou Georgia, Song Kyung, Yen Ivana, Brandhuber Barbara J, Anderson Daniel J, Alvarado Ryan, Ludlam Mary J C, Stokoe David, Gloor Susan L, Vigers Guy, Morales Tony, Aliagas Ignacio, Liu Bonnie, Sideris Steve, Hoeflich Klaus P, Jaiswal Bijay S, Seshagiri Somasekar, Koeppen Hartmut, Belvin Marcia, Friedman Lori S, Malek Shiva (2010). RAF inhibitors prime wild-type RAF to activate the MAPK pathway and enhance growth.. *Nature*.

[16] Heidorn Sonja J, Milagre Carla, Whittaker Steven, Nourry Arnaud, Niculescu-Duvas Ion, Dhomen Nathalie, Hussain Jahan, Reis-Filho Jorge S, Springer Caroline J, Pritchard Catrin, Marais Richard (2010). Kinase-dead BRAF and oncogenic RAS cooperate to drive tumor progression through CRAF.. *Cell*.

[17] Huang Victor, Hepper Donna, Anadkat Milan, Cornelius Lynn (2012). Cutaneous toxic effects associated with vemurafenib and inhibition of the BRAF pathway.. *Archives of dermatology*.

[18] Jakob John A, Bassett Roland L, Ng Chaan S, Curry Jonathan L, Joseph Richard W, Alvarado Gladys C, Rohlfs Michelle L, Richard Jessie, Gershenwald Jeffrey E, Kim Kevin B, Lazar Alexander J, Hwu Patrick, Davies Michael A (2012). NRAS mutation status is an independent prognostic factor in metastatic melanoma.. *Cancer*.

[19] Klein R Matthew, Aplin Andrew E (2009). Rnd3 regulation of the actin cytoskeleton promotes melanoma migration and invasive outgrowth in three dimensions.. *Cancer research*.

[20] Korn Edward L, Liu Ping-Yu, Lee Sandra J, Chapman Judith-Anne W, Niedzwiecki Donna, Suman Vera J, Moon James, Sondak Vernon K, Atkins Michael B, Eisenhauer Elizabeth A, Parulekar Wendy, Markovic Svetomir N, Saxman Scott, Kirkwood John M (2008). Meta-analysis of phase II cooperative group trials in metastatic stage IV melanoma to determine progression-free and overall survival benchmarks for future phase II trials.. *Journal of clinical oncology : official journal of the American Society of Clinical Oncology*.

[21] Lacouture Mario E, Duvic Madeleine, Hauschild Axel, Prieto Victor G, Robert Caroline, Schadendorf Dirk, Kim Caroline C, McCormack Christopher J, Myskowski Patricia L, Spleiss Olivia, Trunzer Kerstin, Su Fei, Nelson Betty, Nolop Keith B, Grippo Joseph F, Lee Richard J, Klimek Matthew J, Troy James L, Joe Andrew K (2013). Analysis of dermatologic events in vemurafenib-treated patients with melanoma.. *The oncologist*.

[22] Larkin James, Ascierto Paolo A, Dréno Brigitte, Atkinson Victoria, Liszkay Gabriella, Maio Michele, Mandalà Mario, Demidov Lev, Stroyakovskiy Daniil, Thomas Luc, de la Cruz-Merino Luis, Dutriaux Caroline, Garbe Claus, Sovak Mika A, Chang Ilsung, Choong Nicholas, Hack Stephen P, McArthur Grant A, Ribas Antoni (2014). Combined vemurafenib and cobimetinib in BRAF-mutated melanoma.. *The New England journal of medicine*.

[23] Long Georgina V, Menzies Alexander M, Nagrial Adnan M, Haydu Lauren E, Hamilton Anne L, Mann Graham J, Hughes T Michael, Thompson John F, Scolyer Richard A, Kefford Richard F (2011). Prognostic and clinicopathologic associations of oncogenic BRAF in metastatic melanoma.. *Journal of clinical oncology : official journal of the American Society of Clinical Oncology*.

[24] Long Georgina V, Stroyakovskiy Daniil, Gogas Helen, Levchenko Evgeny, de Braud Filippo, Larkin James, Garbe Claus, Jouary Thomas, Hauschild Axel, Grob Jean Jacques, Chiarion Sileni Vanna, Lebbe Celeste, Mandalà Mario, Millward Michael, Arance Ana, Bondarenko Igor, Haanen John B A G, Hansson Johan, Utikal Jochen, Ferraresi Virginia, Kovalenko Nadezhda, Mohr Peter, Probachai Volodymyr, Schadendorf Dirk, Nathan Paul, Robert Caroline, Ribas Antoni, DeMarini Douglas J, Irani Jhangir G, Casey Michelle, Ouellet Daniele, Martin Anne-Marie, Le Ngocdiep, Patel Kiran, Flaherty Keith (2014). Combined BRAF and MEK inhibition versus BRAF inhibition alone in melanoma.. *The New England journal of medicine*.

[25] Mandrell Joshua C, Santa Cruz Daniel (2009). Keratoacanthoma: hyperplasia, benign neoplasm, or a type of squamous cell carcinoma?. *Seminars in diagnostic pathology*.

[26] McArthur Grant A, Chapman Paul B, Robert Caroline, Larkin James, Haanen John B, Dummer Reinhard, Ribas Antoni, Hogg David, Hamid Omid, Ascierto Paolo A, Garbe Claus, Testori Alessandro, Maio Michele, Lorigan Paul, Lebbé Celeste, Jouary Thomas, Schadendorf Dirk, O'Day Stephen J, Kirkwood John M, Eggermont Alexander M, Dréno Brigitte, Sosman Jeffrey A, Flaherty Keith T, Yin Ming, Caro Ivor, Cheng Suzanne, Trunzer Kerstin, Hauschild Axel (2014). Safety and efficacy of vemurafenib in BRAF(V600E) and BRAF(V600K) mutation-positive melanoma (BRIM-3): extended follow-up of a phase 3, randomised, open-label study.. *The Lancet. Oncology*.

[27] (2010). Common Terminology Criteria for Adverse Events (CTCAE) Version 4.03. *National Cancer Institute*.

[28] (2013). NCCN Clinical Practice Guidelines in Oncology: Melanoma. Version 1.. *National Comprehensive Cancer Network.*.

[29] Oberholzer Patrick A, Kee Damien, Dziunycz Piotr, Sucker Antje, Kamsukom Nyam, Jones Robert, Roden Christine, Chalk Clinton J, Ardlie Kristin, Palescandolo Emanuele, Piris Adriano, MacConaill Laura E, Robert Caroline, Hofbauer Günther F L, McArthur Grant A, Schadendorf Dirk, Garraway Levi A (2012). RAS mutations are associated with the development of cutaneous squamous cell tumors in patients treated with RAF inhibitors.. *Journal of clinical oncology : official journal of the American Society of Clinical Oncology*.

[30] Poulikakos Poulikos I, Zhang Chao, Bollag Gideon, Shokat Kevan M, Rosen Neal (2010). RAF inhibitors transactivate RAF dimers and ERK signalling in cells with wild-type BRAF.. *Nature*.

[31] Rinderknecht J. D., Goldinger S. M., Rozati S., Kamarashev J., Kerl K., French L. E., Belloni B. (2013). RASopathic skin eruptions during vemurafenib therapy. *PLoS One*.

[32] Satzger Imke, Degen Annette, Asper Hiba, Kapp Alexander, Hauschild Axel, Gutzmer Ralf (2013). Serious skin toxicity with the combination of BRAF inhibitors and radiotherapy.. *Journal of clinical oncology : official journal of the American Society of Clinical Oncology*.

[33] Seghers Amélie Clémentine, Wilgenhof Sofie, Lebbé Céleste, Neyns Bart (2012). Successful rechallenge in two patients with BRAF-V600-mutant melanoma who experienced previous progression during treatment with a selective BRAF inhibitor.. *Melanoma research*.

[34] Siegel R., Ma J., Zou Z., Jemal A. (2014). CA: A Cancer Journal for Clinicians. *Cancer statistics, 2014.*.

[35] Sinha R, Edmonds K, Newton-Bishop J A, Gore M E, Larkin J, Fearfield L (2012). Cutaneous adverse events associated with vemurafenib in patients with metastatic melanoma: practical advice on diagnosis, prevention and management of the main treatment-related skin toxicities.. *The British journal of dermatology*.

[36] Sosman Jeffrey A, Kim Kevin B, Schuchter Lynn, Gonzalez Rene, Pavlick Anna C, Weber Jeffrey S, McArthur Grant A, Hutson Thomas E, Moschos Stergios J, Flaherty Keith T, Hersey Peter, Kefford Richard, Lawrence Donald, Puzanov Igor, Lewis Karl D, Amaravadi Ravi K, Chmielowski Bartosz, Lawrence H Jeffrey, Shyr Yu, Ye Fei, Li Jiang, Nolop Keith B, Lee Richard J, Joe Andrew K, Ribas Antoni (2012). Survival in BRAF V600-mutant advanced melanoma treated with vemurafenib.. *The New England journal of medicine*.

[37] Su Fei, Viros Amaya, Milagre Carla, Trunzer Kerstin, Bollag Gideon, Spleiss Olivia, Reis-Filho Jorge S, Kong Xiangju, Koya Richard C, Flaherty Keith T, Chapman Paul B, Kim Min Jung, Hayward Robert, Martin Matthew, Yang Hong, Wang Qiongqing, Hilton Holly, Hang Julie S, Noe Johannes, Lambros Maryou, Geyer Felipe, Dhomen Nathalie, Niculescu-Duvaz Ion, Zambon Alfonso, Niculescu-Duvaz Dan, Preece Natasha, Robert Lídia, Otte Nicholas J, Mok Stephen, Kee Damien, Ma Yan, Zhang Chao, Habets Gaston, Burton Elizabeth A, Wong Bernice, Nguyen Hoa, Kockx Mark, Andries Luc, Lestini Brian, Nolop Keith B, Lee Richard J, Joe Andrew K, Troy James L, Gonzalez Rene, Hutson Thomas E, Puzanov Igor, Chmielowski Bartosz, Springer Caroline J, McArthur Grant A, Sosman Jeffrey A, Lo Roger S, Ribas Antoni, Marais Richard (2012). RAS mutations in cutaneous squamous-cell carcinomas in patients treated with BRAF inhibitors.. *The New England journal of medicine*.

[38] Sullivan Ryan J, Flaherty Keith T (2013). Resistance to BRAF-targeted therapy in melanoma.. *European journal of cancer (Oxford, England : 1990)*.

[39] Zimmer Lisa, Hillen Uwe, Livingstone Elisabeth, Lacouture Mario E, Busam Klaus, Carvajal Richard D, Egberts Friederike, Hauschild Axel, Kashani-Sabet Mohammed, Goldinger Simone M, Dummer Reinhard, Long Georgina V, McArthur Grant, Scherag André, Sucker Antje, Schadendorf Dirk (2012). Atypical melanocytic proliferations and new primary melanomas in patients with advanced melanoma undergoing selective BRAF inhibition.. *Journal of clinical oncology : official journal of the American Society of Clinical Oncology*.

